# Helminthiasis, eosinophils, COVID-19 and vaccination

**DOI:** 10.4102/sajid.v37i1.423

**Published:** 2022-06-29

**Authors:** Miles B. Markus

**Affiliations:** 1 Wits Research Institute for Malaria, Faculty of Health Sciences, University of the Witwatersrand, Johannesburg, South Africa; 2 School of Animal, Plant and Environmental Sciences, Faculty of Science, University of the Witwatersrand, Johannesburg, South Africa

Helminthiasis, which is characterised inter alia by eosinophilia, is highly prevalent in Africa. What are the implications hereof for susceptibility to coronavirus disease 2019 (COVID-19), progression of the disease and vaccine efficacy? Eosinophilia and eosinopenia are discussed in this context.

For more than 20 years, parasitologists have been researching immune system interactions between helminthiasis and other infections, and the influence of helminthiasis on immunisation against non-helminthic diseases.^1^ Worm infections could have implications for coronavirus disease 2019 (COVID-19) patients, and there are possible consequences for COVID-19 vaccination. In regard hereto, the interesting eosinophil variable (only) is reviewed briefly below. It should be borne in mind, however, that no single factor necessarily explains disease and immunisation outcomes.^2,3^

## Eosinophil biology

Eosinophils are a type of white blood cell and, more specifically, a type of granulocyte. Eosinophil precursors originate in the bone marrow, where eosinophils primarily differentiate and mature, mediated mainly by the cytokine interleukin-5 (IL-5). Eosinophils are then released into the bloodstream and disseminated to other parts of the body. Our understanding of the roles of eosinophils in health and disease is still evolving.^4^

Parasitologically, eosinophilia is a characteristic marker for the T-helper cell type 2 (Th2) immune profile elicited by helminthiasis. Deworming reduces this helminth-associated eosinophilia.^5^

## Eosinophils and COVID-19

Eosinophils are important for an effective immune response to viral pathogens because they attenuate the viral load.^6,7^ Whether this includes severe acute respiratory syndrome coronavirus 2 (SARS-CoV-2) has not been definitively established.^7^ Eosinopenia, interpreted as reflecting impaired innate and adaptive immune responses, has been correlated with severe COVID-19 and fatal outcomes, whereas survivors have been found to exhibit higher eosinophil levels.^8,9^ The pathophysiology of eosinopenia is probably multifactorial, and we do not yet know whether eosinopenia is directly related to the COVID-19 disease process.^7^

Having a Th2-asthma profile could be an important predictive factor for reduced COVID-19 severity,^10^ although the matter is still being debated.^11^ An important question arises: is eosinophilia associated with the Th2-helminthiasis immune profile likewise protective in COVID-19 patients? We cannot at this stage assume that the answer is ‘Yes’, because of current uncertainty, in the context of viral diseases, as to how comparable eosinophils are in asthmatics and non-asthmatics.^11^

## Eosinophils and anti-COVID-19 vaccination

There is a need to demonstrate whether SARS-CoV-2 vaccines worsen eosinophil-associated disease by causing eosinophil-associated immunopotentiation.^6^ Such aggravation could be problematic. The reason why the possibility should be investigated is that it occurred in animal studies^6^ when exposure to the SARS-CoV-1 virus followed anti-SARS-CoV-1 vaccination (note that SARS-CoV-1 and SARS-CoV-2 are closely related).

## Conclusion

Eosinophil-associated considerations regarding COVID-19 are emerging. After two years of the COVID-19 pandemic, we still do not understand the implications of concomitant helminthiasis in persons who contract COVID-19 infection, or the implications of helminthiasis for anti-COVID-19 immunisation. Accordingly, these are topics for future research, especially the consequences of the helminth-induced eosinophilia that is so prevalent in human populations in developing countries,^2,5^ where severe and fatal cases of COVID-19 have arguably been less numerous overall than anticipated.
